# Immune Regulation of RAW264.7 Cells *In Vitro* by Flavonoids from *Astragalus complanatus* via Activating the NF-*κ*B Signalling Pathway

**DOI:** 10.1155/2018/7948068

**Published:** 2018-04-05

**Authors:** Yu Li, Ning Hao, Suping Zou, Tingting Meng, Huanqing Tao, Pengfei Ming, Manman Li, Hongyan Ding, Jinchun Li, Shibin Feng, Xichun Wang, Jinjie Wu

**Affiliations:** College of Animal Science and Technology, Anhui Agricultural University, Hefei 230036, China

## Abstract

The current study aimed at investigating the effects of flavonoids from *Astragalus complanatus* (FAC) on the proliferation, the contents, and gene expression levels of cytokines, secretion of surface stimulating factors, cell cycle, and the expression level of the NF-*κ*B signalling pathway in RAW264.7 cells. Our results revealed that compared with control group, the contents of IL-6, IL-1*β*, TNF-*α*, and NO and the mRNA expression levels of IL-6, IL-1*β*, TNF-*α*, and iNOS in FAC-treated groups significantly increased (*p* < 0.01). Moreover, FAC induced macrophage activation to release the above-mentioned mediators partly involved in NF-*κ*B/MAPK signalling pathways. Therefore, FAC regulates immune function in RAW264.7 cells via activating the NF-*κ*B signalling pathway. FAC could be applicable for agriculture, drug research, and food industry as a potent immune-modulatory agent.

## 1. Introduction

As a traditional Chinese medicine, *Astragalus* regulates the immune response via regulating macrophage inflammatory response and activating heparanase [1, 2]. *Astragalus* contains flavonoids, saponins, polysaccharides, and trace elements and has a role to hepatoprotective, cardiac, antiaging, anticancer, and anti-inflammatory effect [3]. *Astragalus* and its effective components *Astragalus* flavonoids have immune enhancement effects and play an important role in promoting antibody production, regulation of immunity, antivirus, and antibacterial [4]. Xu et al. have confirmed that flavonoids from *Astragalus complanatus* (FAC) can improve the function of mouse mononuclear macrophages, inhibit delayed-type hypersensitivity in mice, and promote the proliferation of mouse spleen lymphocytes [5, 6]. However, the exact underlying mechanism of immune action for *Astragalus* is unknown, thereby hampering its development [7].

The production of nitric oxide (NO) and the secretion of cytokines such as interleukin-1*β* (IL-1*β*), interleukin-6 (IL-6), and tumor necrosis factor (TNF-*α*) in RAW264.7 cells are indicators of immune function [8]. Moreover, previous studies have shown that CD40, CD80, and CD86 contribute to the immunopotentiating action on dendritic cell (DC) surfaces, which is induced by polysaccharide [9].

In recent years, the pharmacological effects of *Astragalus* and its mechanism have been studied extensively. However, there is no report about the role of NF-*κ*B signalling pathways in regulating the immune function of flavonoids in *Astragalus membranaceus*. Therefore, the signal transduction mechanism of *Astragalus membranaceus* can be elucidated by studying the effect of astragalus flavonoids in Radix Astragali on RAW264.7 cells from NF-*κ*B signalling transduction pathway, which lays a theoretical foundation for the clinical application of *Astragalus.*

## 2. Materials and Methods

### 2.1. Reagents and Chemicals

Flavonoids (purity > 98%) from *Astragalus complanatus* was purchased from the National Institute for the Control of Pharmaceutical and Biological Products (Beijing, China). A Cell Counting Kit-8 (CCK-8) was purchased from Dojindo Molecular Technology Inc. (Gaithersburg, MD). A cell cycle kit and ELISA kits specific for mouse TNF-*α*, IL-6, IL-1*β*, and NO were obtained from Senbeijia Biological Inc. (Nanjing, China). TRIzol, First-strand cDNA Synthesis Kit, and a quantitative PCR kit were obtained from Takara (Dalian, China). The primary anti-*β*-actin, p50, p65, and p-p65 antibodies and horseradish peroxidase-conjugated rabbit anti-mouse secondary antibodies were purchased from Beyotime Institute of Biotechnology (Jiangsu, China). The pacific blue anti-mouse CD40, PE anti-mouse CD80, and FITC anti-mouse CD86 antibodies and the corresponding isotype controls were purchased from BioLegend (CA, USA). All other chemicals were of analytic grade (Sinopharm Chemical Reagent Co. Ltd., China).

### 2.2. RAW264.7 Culture and Treatment

RAW264.7 cells were cultured under condition of 37°C and 5% CO_2_ in high-glucose Dulbecco's Modification of Eagle's Medium (DMEM) (HyClone, Logan, UT) with 10% fetal bovine serum (FBS) (Clark Bioscience, Seabrook, MD, USA) [3]. The cells were passaged in 80% confluent culture flask, and the cells were observed every 48 h. The FAC-treated group are the following: 0 *μ*g/mL FAC-treated group, 0.1 *μ*g/mL FAC-treat group, 1 *μ*g/mL FAC-treated group, 10 *μ*g/mL FAC-treated group, 5 *μ*mol/L PDTC (NF-*κ*B inhibitor), and 10 *μ*g/mL FAC+ 5 *μ*mol/L PDTC. After cells were pretreated with PDTC for 1 h, FAC was added for 24 h.

### 2.3. Optimum Concentration of FAC Treatment

Cells in logarithmic growth phase were adjusted to a concentration of 1 × 10^5^ cells/mL. Cells were seeded in 96-well plates at 100 *μ*L per well. After 24 hours of culture, cells were incubated at final concentrations of 0, 0.1, 1, and 10 *μ*g/mL of FAC, respectively, and set the blank control group. After incubation for 24 h, 10 *μ*L of CCK-8 solution was added to the control wells and FAC-treated wells. After incubation for 3 h, the absorbance at 450 nm was measured using a microplate reader (Thermo Multiskan MK3, USA).

### 2.4. CCK-8 Cell Viability

Cells were seeded in 96-well plates at a density of 1 × 10^5^ cells/mL in a logarithmic growth phase. The plates were incubated at 37°C and 5% CO_2_ for 24 h. Then, 10 *μ*L of CCK-8 solution was added to each well, and cells were incubated for an additional 4 h. The optical density (OD) was measured at 450 nm by using a plate reader (Thermo Multiskan MK3, USA).

### 2.5. ELISA Assay

RAW264.7 cells were seeded in 6-well plates (1 × 10^5^ cells/mL) and treated with various concentrations (0–10 *μ*g/mL) of FAC with or without 10 *μ*L PDTC. Cells were centrifuged at 4500 rpm for 10 min, and the concentrations of IL-1*β*, IL-6, TNF-*α*, and NO were measured in the cell supernatants with a mouse ELISA kit, according to the manufacturer's instructions (Senbeijia Biological Inc., Nanjing, China).

### 2.6. Quantitative Real-Time Polymerase Chain Reaction (qRT-PCR)

Total RNA was isolated from RAW264.7 cells using TRIzol reagent, and RNA was reverse-transcribed into cDNA with a Toyobo First Strand cDNA Synthesis Kit. Relative mRNA expression levels were detected by using a Biomiga SYBR qPCR mix kit and ABI 7500 qRT-PCR. Data are presented as 2^−△△ct^. The primer sequences of IL-1*β*, IL-6, TNF-*α*, iNOS, and GAPDH that were used for qRT-PCR were presented in Table 1.

### 2.7. Evaluation of CD40, CD80, and CD86 Expression

Cells were collected after they were treated with different concentrations (0–100 *μ*g/mL) of FAC in the presence or absence of PDTC for the indicated times. The cell surfaces were blocked with 15% sheep serum at 4°C for 15 min and were then washed twice with phosphate buffer solution (PBS, pH 7.2). Then, cells were incubated with monoclonal antibodies against CD40, CD80, CD86, and the corresponding fluorescent markers for 30 min at 4°C. After cells were washed twice with PBS and resuspended in PBS, they were subjected to flow cytometry with a FAC Scan platform (Becton Dickinson).

### 2.8. Flow Cytometry Analysis of Cell Cycle Regulation

RAW264.7 cells (1 × 10^5^ cells/mL) were seeded in 6-well plates and treated with various concentrations (0–10 *μ*g/mL) of FAC in the presence or absence of PDTC for 24 h. Then, cells were collected, washed once with cold PBS, fixed in 75% cold alcohol overnight at 4°C, and washed twice with cold PBS. Fixed cells were resuspended in 100 *μ*L RNase at 37°C and incubated with 400 *μ*L propidium iodide (PI) at 4°C in a dark room for 30 min. Cell cycle progression was analyzed by using flow cytometry with a FAC Scan platform (Becton Dickinson).

### 2.9. Western Blotting Assay

After cells were cultured for the indicated times, they were collected and washed twice with cold PBS at 800 g for 10 min. Total protein was extracted by using RIPA buffer. Protein concentrations were determined by using the BCA method as previous report described [10–12]. Proteins (50 *μ*g) were separated through 10% SDS-PAGE and subsequently transferred to PVDF membranes. The membranes were blocked with 5% bovine serum albumin (BSA) containing 0.05% Tween-20 in TBST for 4 h at room temperature and were then incubated with the primary antibodies overnight at 4°C. The membranes were washed twice and incubated with the secondary antibodies for 1 h at room temperature. The protein bands were detected with a chemiluminescence Western blotting detection system.

### 2.10. Statistical Analysis

Data were analyzed with the SPSS software package 17.0 (SPSS Inc., Chicago, IL, USA) and are presented as the mean ± standard deviation (SD). The differences among experimental groups were analyzed by using one-way ANOVA. Compared with the control group, *p* values below 0.05 were considered to be statistically significant (^∗^*p* < 0.05, ^∗∗^*p* < 0.01). Compared with the 10 *μ*g/mL FAC-treated group, *p* values below 0.05 were considered to be statistically significant (^#^*p* < 0.05, ^##^*p* < 0.01).

## 3. Results

### 3.1. Optimum Concentration of FAC Treatment

**Figure 1:**
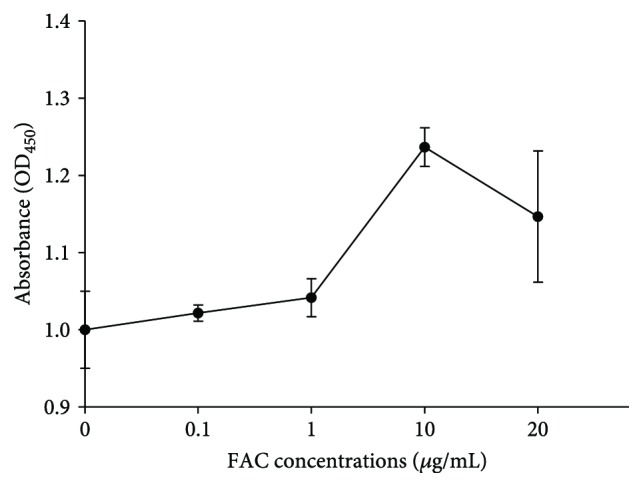
Effects of various concentrations of FAC on RAW264.7 cell proliferation rate. Data are expressed as mean ± SD with five replications in each treatment.

As showed in Figure 1, RAW264.7 cells were treated with different concentrations of FAC (0–20 *μ*g/mL). At the concentration range of 0–10 *μ*g/mL, cell viability increased with the increase of FAC concentrations and reached the maximum at 10 *μ*g/mL. When the concentration of FAC was 10–20 *μ*g/mL, the cell viability decreased with the increase of FAC concentrations. Therefore, 10 *μ*g/mL FAC was selected as the optimum concentration for subsequent experiments.

### 3.2. Effect of FAC on the Cell Viability

**Figure 2:**
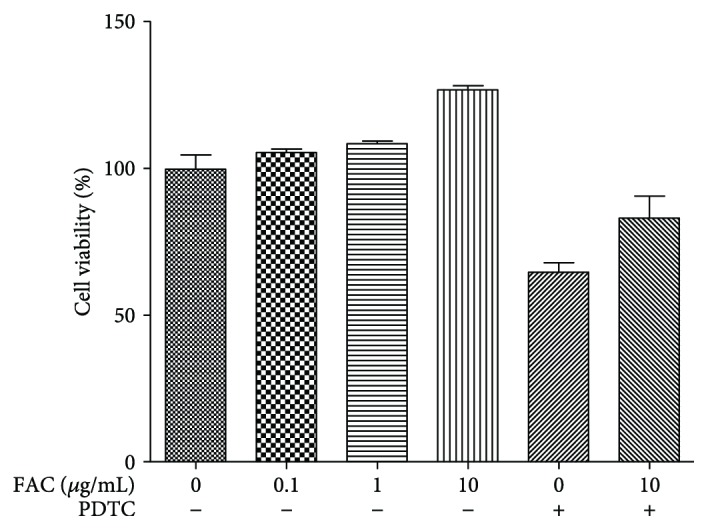
Effect of various concentrations of FAC on cell viability. RAW264.7 cells were treated with different concentrations of FAC (0–10 *μ*g/mL) in the presence or absence of 10 *μ*mol/mL PDTC. ∗ represents a significant difference (*p* < 0.05); ∗∗ represents a markedly significant difference versus the control group. # represents a significant difference (*p* < 0.05); ## represents a markedly significant difference (*p* < 0.01) versus the 10 *μ*g/mL FAC-treated group. Data are expressed as mean ± SD with five replications in each treatment.

As showed in Figure 2, compared with the control group, 0.1–10 *μ*g/mL FAC could increase the activity of the cells to some extent. After adding PDTC alone or combination with 10 *μ*g/mL FAC, cell viability was reduced, but the differences were not significant (*p* > 0.05).

### 3.3. FAC Dose Dependently Increased IL-1*β*, IL-6, TNF-*α*, and NO Expression in RAW264.7 Cells

**Figure 3:**
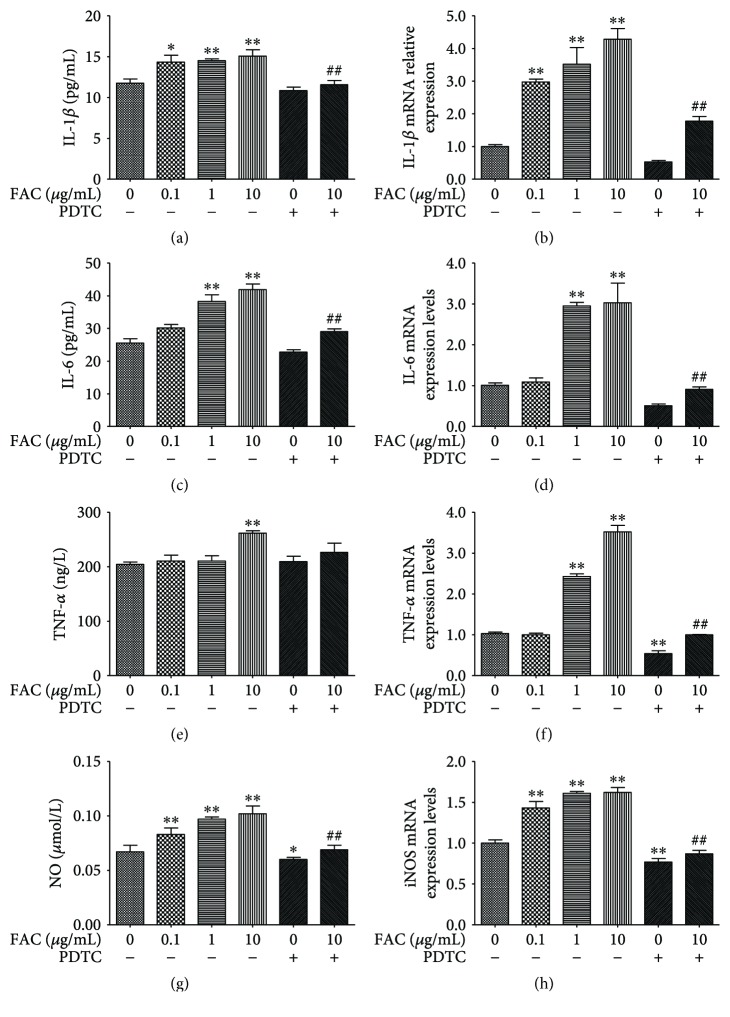
Effect of FAC on cytokines, NO content, and gene expression. RAW264.7 cells were treated with 0–10 *μ*g/mL FAC in the absence or presence of 10 *μ*mol/mL PDTC. The content (a) and mRNA levels (b) of IL-1*β*; content (c) and mRNA levels (d) of IL-6; content (e) and mRNA levels (d) of TNF-*α*; content of NO (g) and mRNA levels of iNOS (h). ^∗^*p* < 0.05, ^∗∗^*p* < 0.01 versus the control group. ^##^*p* < 0.01 versus the 100 *μ*g/mL FAC-treated group. Data are expressed as mean ± SD with 9 replications in one treatment.

As showed in Figure 3, compared with the control group, the content of IL-1*β* significantly increased (*p* < 0.05) and the content of NO increased significantly (*p* < 0.01) in 0.1 *μ*g/mL FAC-treated groups. The content of IL-1*β*, IL-6, and NO markedly increased (*p* < 0.01) in 1 and 10 *μ*g/mL FAC-treated groups. The content of TNF-*α* significantly increased in 10 *μ*g/mL FAC-treated groups (*p* < 0.01). The content of NO significantly decreased (*p* < 0.05) in PDTC treatment groups. The levels of IL-1*β*, IL-6, and NO in 10 *μ*g/mL FAC + PDTC group are significantly lower than those in 10 *μ*g/mL FAC groups (*p* < 0.01). Compared with the control group, the expression of IL-1*β* and iNOS mRNA significantly increased in 0.1–10 *μ*g/mL FAC-treated groups (*p* < 0.01). The expression of IL-6 and TNF-*α* mRNA expression markedly decreased (*p* < 0.01) in 1 and 10 *μ*g/mL FAC-treated groups (*p* < 0.01). The expression of TNF-*α* and iNOS mRNA in PDTC-treated group is significantly lower than that in control groups (*p* < 0.01). The expression of IL-1*β*, IL-6, TNF-*α*, and iNOS mRNA significantly decreased (*p* < 0.01) in 10 *μ*g/mL FAC + PDTC groups compared with that in 10 *μ*g/mL FAC group.

### 3.4. Evaluation of CD40, CD80, and CD86 Expression

**Figure 4:**
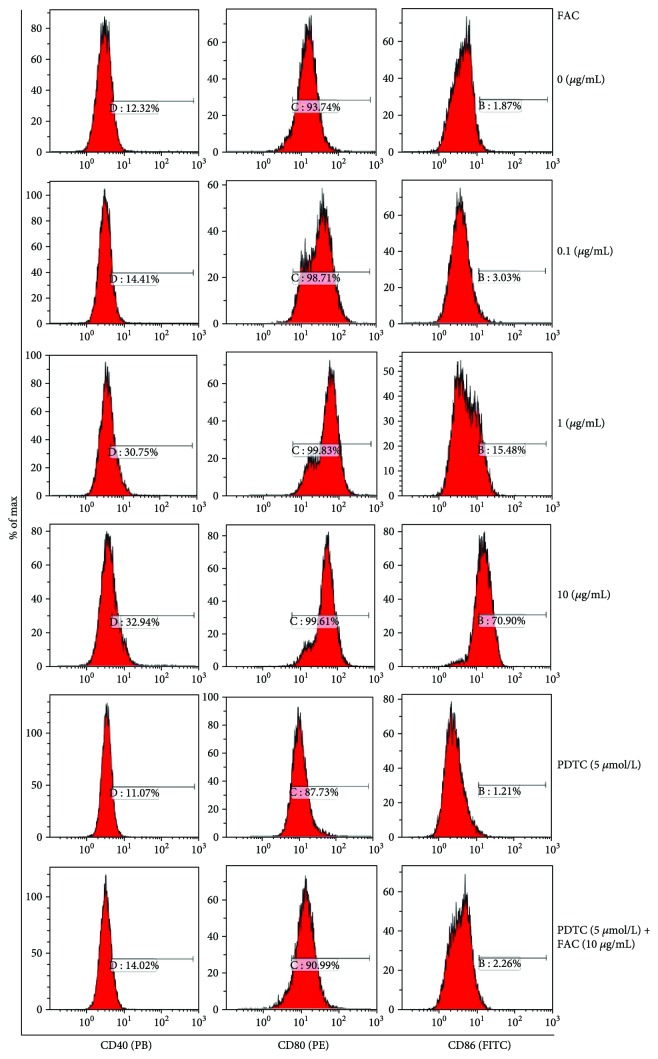
Effect of FAC on the secretion of CD40, CD80, and CD86. RAW264.7 cells were treated with different concentrations (0–10 *μ*g/mL) of FAC in the absence or presence of 10 *μ*mol/mL PDTC. From the left to right, secretion of CD40 (PB), CD80 (PE), and CD86 (FITC). After the cell surface factor was detected by flow cytometry, the results were expressed by histogram.

As showed in Figure 4, FAC exerts different effects on CD40, CD80, and CD86 secretion in RAW264.7 cells. Compared with the control group, the secretion of CD40 increased from 14.41% to 32.94% with the increase of FAC concentrations, and the secretion of CD40 decreased to 11.07% after addition of inhibitor PDTC. Compared with 10 *μ*g/mL FAC group, the secretion of CD40 in 10 *μ*g/mL FAC + PDTC treatment group was reduced to 14.02%. The expression of CD80 showed no significant changes after RAW264.7 cells were treated with various concentrations of FAC. Compared with the control group, the secretion of CD86 increased from 3.03% to 70.90% with the increase of FAC concentrations, and the secretion of CD86 decreased to 1.21% in the PDTC treatment group. The secretion of CD86 decreased to 2.26% in 10 *μ*g/mL FAC + PDTC treatment group compared with 10 *μ*g/mL FAC.

### 3.5. FAC Promoted Cell Proliferation by Inducing Cell Cycle in RAW264.7 Cells

**Figure 5:**
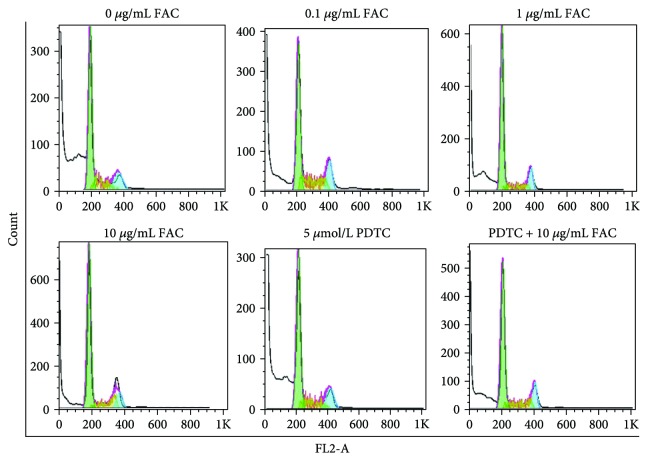
Effect of FAC on cell cycle in RAW264.7 cells. All cells (adherent and nonadherent) were collected and analyzed by flow cytometry.

As showed in Figure 5 and Table 2, compared with the control group, the ratio of G2/M phase increased with the increase of FAC concentrations. PDTC inhibits the ratio of G2/M phase. However, the changes are relatively small. Therefore, different concentrations of FAC and PDTC had no significant effect on cell proliferation.

### 3.6. FAC Inhibited the NF-*κ*B Signalling Pathway-Related Key Protein Expression in RAW264.7 Cells

**Figure 6:**
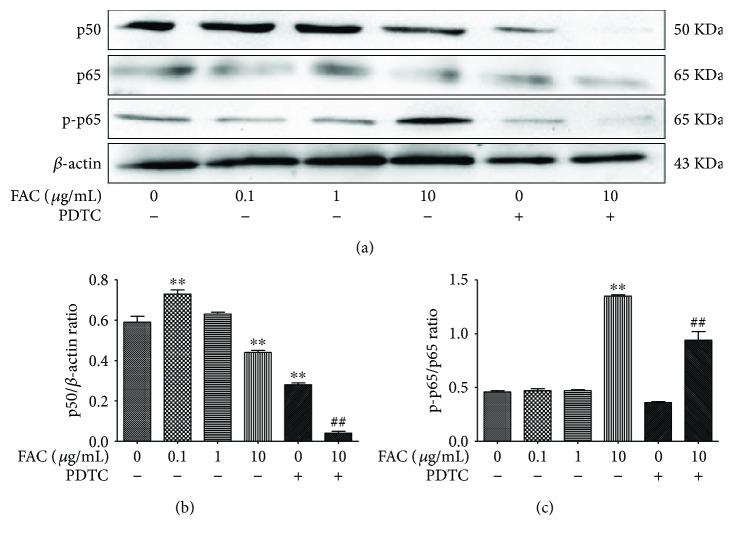
Effects of FAC on the key molecules in NF-*κ*B signalling pathway. (a) Protein expression levels of p50, p65, p-p65, and *β*-actin; (b) the ratio of p50/*β*-actin; (c) the ration of p-p65/p65. ^∗∗^*p* < 0.01 versus the control group. ^##^*p* < 0.01 versus the 100 *μ*g/mL FAC-treated group. Data are expressed as mean ± SD with 3 replications in one treatment.

As showed in Figures 6(a) and 6(b), the protein of p50 and the ratio of p50/*β*-actin in 0.1 and 10 *μ*g/mL FAC treatment groups were significantly higher than those in the control group (*p* < 0.01). The protein of p50 and the ratio of p50/*β*-actin further significantly decreased in PDTC treatment group (*p* < 0.01). Compared with 10 *μ*g/mL FAC treatment group, the protein of p50 and the ratio of p50/*β*-actin significantly reduced (*p* < 0.01) in 10 *μ*g/mL FAC + PDTC treatment group. Compared with the control group, the p-p65/p65 value was significantly increased (*p* < 0.01) in the 10 *μ*g/mL FAC-treated group compared with the control group. The protein of p65 and the ratio of p-p65/p65 was significantly higher in 10 *μ*g/mL FAC-treated group than that in the control group. The protein of p65 and the ratio of p-p65/p65 significantly increased in the 10 *μ*g/mL FAC + PDTC group (*p* < 0.01).

## 4. Discussion

Previous studies have showed that FAC has the function of free radical scavenging, resistance to body mutations, the protection of the liver function, anti-myocardial ischemia, reduction of the inflammatory response, and immunity enhancement [3, 13, 14]. The beneficial biological function of FAC makes it possible to apply to clinical disease prevention and treatment. Other reports have showed that flavonoids can reduce the LPS-induced RAW264.7 inflammatory cells by reducing NO secretion [15]. The results of this study show that FAC has a significant effect on IL-1*β*, IL-6, TNF-*α*, iNOS secretion, and gene expression in RAW264.7 cells, and the secretion of cytokines and expression of NF-*κ*B pathway were decreased after treated with inhibitor PDTC which indicated that FAC and PDTC can affect the secretion of cytokines and gene expression.

CD40, CD80, and CD86 react with the antigen-presenting effect of immune cells and play a costimulatory role in heart transplantation. The surface costimulatory factors have a protective effect on the immunological activity of immune cells and induce the expression of cytokines and other functions [16–18]. In our study, the secretion of CD40 and CD86 increased in RAW264.7 cells treated with FAC. The secretion of CD80 showed no significant change. These results suggested that CD40 and CD86, not CD80, are involved in the immune regulation of RAW264.7 cells by FAC.

The changes of cell from diploid to tetraploid stage are the performance of cell division and proliferation. Cells in the G1 period (period of preparation) are preparing for the presynthesis of RNA and ribosome. S period spanning cycle analysis of a peak in the lower peak and the larger spans is the synthesis of DNA. Studies have showed that flavonoids have the effect of inducing apoptosis, inhibiting the proliferation of human tumor cells, and stimulating the activation of mast cells [19–21]. In the present study, the activity of the cells was gradually enhanced with the increase of FAC concentration, but the cell activity was significantly decreased after the addition of the inhibitor. It can be seen that FAC can increase cell activity and promote cell proliferation.

FAC enhances the cytotoxic activity of NK cells, improves the synergistic vaccine resistant to the infection of the virus, stimulates the intestinal tract, and enhances the function of the intestinal tract [22–24]. It has also been reported that FAC significantly inhibits the expression of extracellular signal-regulated kinases (ERK), p38, and JNK phosphorylated proteins by signal transduction and inhibits NF-*κ*B p65 transformation. LPS-induced RAW264.7 cells can inhibit inflammatory cytokines by blocking NF-*κ*B and MAPK signalling pathway [25, 26]. In this study, low concentration of FAC can increase the NF-*κ*B pathway-related protein, while p50 protein expression decreased in other groups. The ratio of p-p65/p65, as the FAC concentrations increased and reached the maximum in 10 *μ*g/mL. The ratio of p-p65/p65 was significantly lower in the 10 *μ*g/mL FAC + PDTC group than that in the high-concentration group, which indicated that FAC promoted phosphorylation of p65 protein, through the NF-*κ*B signalling pathway, to promote immune function of RAW264.7 cells.

In summary, these results show that FAC promoted the expression of IL-6, IL-1*β*, TNF-*α*, and iNOS gene and phosphorylation of p65 protein and the secretion of CD40 and CD86 on the cell surface through NF-*κ*B signalling transduction pathway, thereby increasing the activity of cells to enhance the immune function.

## Figures and Tables

**Table 1:** Primer sequences used in qRT-PCR.

Genes	Accession numbers	Primer sequence (5→3′)	Product size (bp)
IL-1*β*	NM_008361.3	Forward primer GCCACCTTTTGACAGTGATGAG	165
		Reverse primer AGTGATACTGCCTGCCTGAAG	
IL-6	NM_031168.1	Forward primer CAACGATGATGCACTTGCAGA	201
		Reverse primer TCTCTCTGAAGGACTCTGGCT	
TNF-*α*	NM_001278601.1	Forward primer ACCTGGCCTCTCTACCTTGT	161
		Reverse primer CCCGTAGGGCGATTACAGTC	
iNOS	NM_010927.4	Forward primer AGGGACTGAGCTGTTAGAGACA	156
		Reverse primer AAGAGAAACTTCCAGGGGCAAG	
GAPDH	NM_001289726.1	Forward primer GGTGAAGGTCGGTGTGAACG	232
		Reverse primer CCCGTAGGGCGATTACAGTC	

**Table 2:** Effect of FAC on the cell cycle.

Concentration Cell cycle	FAC (*μ*g/mL)				PDTC (5 *μ*mol/L)	PDT (5 *μ*mol/L) + 10 *μ*g/mLFAC
	0	0.1	1	10		
RMS	25.86	19.99	13.42	14.46	27.55	15.27
Freq.G1	24.75	27.4	45.72	55.66	26.42	40.78
Freq.S	5.89	11.76	8.67	12.82	6.01	9.55
Freq.G2	6.92	8.12	8.15	9.93	6.79	7.87
G1 mean	194	208	199	185	210	206
G2 mean	367	407.23	377.97	370	412	404
G1 cv	7.85	7.8	7.15	8.77	8.9	8.36
G2 cv	10.95	5.8	4.7	7.62	8.78	4.5
Freq.sub-G1	59.36	34.33	36.71	16.66	57.53	30.06
Freq.sub-G2	0.06	3.74	1.25	0.89	0.41	1.84

## Abbreviation

FAC: Flavonoids from *Astragalus complanatus*
NO: Nitric oxide
IL-1*β*: Interleukin-1*β*
IL-6: Interleukin-6
TNF-*α*: Tumor necrosis factor
DC: Dendritic cell
CCK-8: Cell Counting Kit-8
FBS: Fetal bovine serum
PDTC: Pyrrolidinedithiocarbamic acid
OD: Optical density
qRT-PCR: Quantitative real-time polymerase chain reaction
PBS: Phosphate buffer solution.
